# Access to Care and Prevalence of Hypertension and Diabetes Among Syrian Refugees in Northern Jordan

**DOI:** 10.1001/jamanetworkopen.2020.21678

**Published:** 2020-10-14

**Authors:** Ruwan Ratnayake, Fatma Rawashdeh, Raeda AbuAlRub, Nahla Al-Ali, Muhammad Fawad, Mohammad Bani Hani, Ravi Goyal, P. Gregg Greenough, Khaldoun Al-Amire, Rowaida AlMaaitah, Parveen Parmar

**Affiliations:** 1 International Rescue Committee, Amman, Jordan; 2 Department of Infectious Disease Epidemiology, London School of Hygiene and Tropical Medicine, London, United Kingdom; 3 Department of Community and Mental Health Nursing, Jordan University of Science and Technology, Irbid, Jordan; 4 Mathematica Policy Research Inc, Princeton, New Jersey; 5 Department of Global Health and Population, Harvard T.H. Chan School of Public Health, Boston, Massachusetts; 6 Division of Global Emergency Medicine, University of Southern California, Los Angeles

## Abstract

**Importance:**

The management of noncommunicable diseases in humanitarian crises has been slow to progress from episodic care. Understanding disease burden and access to care among crisis-affected populations can inform more comprehensive management.

**Objective:**

To estimate the prevalence of hypertension and diabetes with biological measures and to evaluate access to care among Syrian refugees in northern Jordan.

**Design, Setting, and Participants:**

This cross-sectional study was undertaken from March 25 to April 26, 2019, in the districts of Ramtha and Mafraq, Jordan. Seventy clusters of 15 households were randomly sampled, and chain referral was used to sample Syrian households, representative of 59 617 Syrian refugees. Adults were screened and interviewed about their access to care. Data analysis was performed from May to September 2019.

**Exposures:**

Primary care delivered through a humanitarian organization since 2012.

**Main Outcomes and Measures:**

The main outcomes were self-reported prevalence of hypertension and diabetes among adults aged 18 years or older and biologically based prevalence among adults aged 30 years or older. The secondary outcome was access to care during the past month among adults aged 18 years or older with a diagnosis of hypertension or diabetes.

**Results:**

In 1022 randomly sampled households, 2798 adults aged 18 years or older, including 275 with self-reported diagnoses (mean [SD] age, 56.5 [13.2] years; 174 women [63.3%]), and 915 adults aged 30 years or older (608 women [66.5%]; mean [SD] age, 46.0 [12.8] years) were screened for diabetes and hypertension. Among adults aged 18 years or older, the self-reported prevalence was 17.2% (95% CI, 15.9%-18.6%) for hypertension, 9.8% (95% CI, 8.6%-11.1%) for diabetes, and 7.3% (95% CI, 6.3%-8.5%) for both conditions. Among adults aged 30 years or older, the biologically based prevalence was 39.5% (95% CI, 36.4%-42.6%) for hypertension, 19.3% (95% CI, 16.7%-22.1%) for diabetes, and 13.5% (95% CI, 11.4%-15.9%) for both conditions. Adjusted for age and sex, prevalence for all conditions increased with age, and women had a higher prevalence of diabetes than men (adjusted prevalence ratio, 1.3%; 95% CI, 1.0%-1.7%), although the difference was not significant. Complications (57.4%; 95% CI, 51.5%-63.1%) and obese or overweight status (82.8%; 95% CI, 79.7%-85.5%) were highly prevalent. Among adults aged 30 years or older with known diagnoses, 94.1% (95% CI, 90.9%-96.2%) currently took medication. Among adults aged 18 years or older with known diagnoses, 26.8% (95% CI, 21.3%-33.1%) missed a medication dose in the past week, and 49.1% (95% CI, 43.3%-54.9%) sought care in the last month.

**Conclusions and Relevance:**

During this protracted crisis, obtaining care for noncommunicable diseases was feasible, as demonstrated by biologically based prevalence that was only moderately higher than self-reported prevalence. The high prevalence of complications and obese or overweight status, however, suggest inadequate management. Programs should focus on reinforcing adherence and secondary prevention to minimize severe morbidity.

## Key Points

**Question:**

What is the prevalence of hypertension and diabetes among long-displaced Syrian refugees in northern Jordan and what is their level of access to care?

**Findings:**

In this cross-sectional study of 1022 randomly sampled households of Syrian refugees, the biologically based prevalence of hypertension and diabetes was moderately higher than self-reported prevalence. Among the participants, 57.4% had 1 or more complication, 82.8% were obese or overweight, 49.1% sought care in the past month, and 26.8% missed their medications in the past week.

**Meaning:**

These findings suggest that long-term disease management is inadequate, in that Syrian refugees were generally aware of their diagnoses and had access to medication, but complications and factors associated with severe disease were highly prevalent.

## Teaser

This cross-sectional study estimates the prevalence of hypertension and diabetes with biological measures and evaluates access to care among Syrian refugees in northern Jordan.

## Introduction

In the Eastern Mediterranean region, the transition from a burden of primarily infectious diseases to noncommunicable diseases (NCDs) has been associated with increased population growth and longevity.^1,2^ Proportional mortality from NCDs has been projected to increase from 62% in 2015 to 70% in 2030.^3,4^ Conflicts in Iraq, Syria, and Yemen have made the inadequate management of NCDs among conflict-affected and displaced populations a major public health issue.^5^ NCD management in humanitarian settings is poorly studied, and health responses have been slow to move away from the paradigm of episodic clinical care.^5,6,7^ Health systems and humanitarian organizations are challenged to provide integrated and cost-effective approaches to stabilize acute presentations, ensure continuous treatment, provide access to medications and insulin, provide patient education, and manage acute complications.^5,7^

Specifically, the crisis in Syria has greatly impacted regional health trends and national health systems.^1^ As of January 2020, 5.6 million refugees were displaced to Turkey, Lebanon, Jordan, and Iraq.^8^ In Jordan, 1 in 14 people is a registered refugee, and 79% of refugees live outside camps in urban and periurban areas.^8,9^ Household surveys have documented that one-half of refugee households have 1 or more adult with an NCD.^10^ A 2016 household survey among Syrians in northern Jordan found the most prevalent diagnoses to be hypertension (14.0%) and diabetes (9.2%).^11^ A 2015 clinic-based survey among Syrian individuals with diabetes in Bekaa Valley, Lebanon, found that 30% of patients received a diagnosis during displacement, decreasing the likelihood that they had received comprehensive education on disease management.^12^ Because Syrian refugees may have developed NCDs after an extended displacement and may lack a diagnosis and awareness of their condition, it follows that neither the disease burden nor health care utilization is well-understood.

Syrian refugees in Jordan access primary care from clinics run by the Jordanian Ministry of Health, nongovernmental organizations (NGOs), and the private sector. In January 2018, facing budget shortfalls, the Ministry of Health reduced subsidies for refugees at public clinics (reinstated in March 2019).^13,14^ Household surveys have cited costs, lack of knowledge of services, and availability of services as primary barriers to NCD care.^10,11,13^ Interruptions likely affect disease control; in 2016, 25% of surveyed patients with NCDs in northern Jordan reported medication interruptions longer than 2 weeks during the past 6 months, primarily because of costs.^11^

There is emerging evidence that community health worker (CHW) models that focus on NCDs can facilitate linkage and continuity of care.^15,16,17,18^ The International Rescue Committee, a humanitarian organization that has provided primary care for Syrian refugees since 2012, has integrated community health into the primary care model. As part of a study to design and evaluate a CHW model for the management of NCDs among refugees, we conducted a household survey among Syrian refugees living outside camps in northern Jordan. The primary objectives were to quantify prevalence using biological measures of hypertension and diabetes, determine the proportion of known and unknown diagnoses, and evaluate access to care for diabetes and hypertension in the catchment area.

## Methods

### Study Design, Setting, and Participants

Ethical approval for this cross-sectional study was granted from the institutional review boards of the International Rescue Committee, Jordan University of Science and Technology, and University of Southern California. Written informed consent was obtained from participants. The findings are reported according to the Strengthening the Reporting of Observational Studies in Epidemiology (STROBE) reporting guideline for cross-sectional studies.

A 2-stage cluster survey was performed from March 25 to April 26, 2019, in the 11 administrative areas of urban Ramtha District (Irbid Governorate) and Mafraq Qasabah District (Mafraq Governorate), which host refugees originating from southern Syria. Covering a registered population of 59 617 refugees living outside of camps, the survey included 88% of the registered refugee population (data supplied by the United Nations High Commissioner for Refugees).^19^ Study participants were adult, nonpregnant refugees aged 18 years or older.

### Cluster Development, Sample Size, and Sampling

Because no database of refugee households was available, a cluster sampling design was used. To construct clusters, a grid was superimposed over maps developed in Quantum GIS software version 3.6.0 (Open Source Geospatial Foundation Project) using shape files created with the LandScan database and a Google Satellite layer.^11^ The areas within the grid borders defined a cluster (see the Figure).^20,21^

**Figure.**
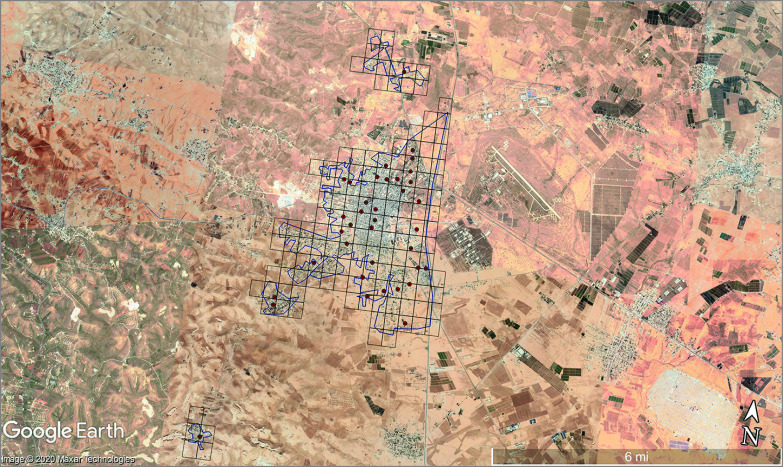
Spatial Cluster Sampling Approach, Mafraq Qasabah District, Jordan The areas within the grid borders defined a cluster, and dots represent the sampled geospatial coordinate that started the chain referral process. Google Satellite source information: main map (imagery date: May 6, 2020; 32°22′10.62” N 36°10′04.83” E, elevation 0 ft [0 km], eye altitude 14.63 mi [23.41 km]).

To detect the prevalence of self-reported hypertension and diabetes among adults (aged ≥18 years) (23% and 16% respectively, based on averaging estimates from surveys in northern Jordan^11^ and precrisis Syria^22^) such that the 95% CI had a precision of 5%, a sample size of 1050 households was calculated. A design effect of 1.5, 10% nonresponse rate, and 2 adults aged 18 years or older per household were assumed.^11^ A large number of clusters relative to households per cluster (70 clusters of 15 households) was used to increase the likelihood of finding sufficient refugee households embedded within host communities.

Sampling occurred in 2 stages: sampling of clusters and households within clusters. To select clusters, 70 geospatial coordinates were randomly allocated across the administrative areas, proportional to the population size of refugees. A cluster was sampled if a coordinate fell within its boundary. To sample households within a cluster, community health volunteers first located the household, shop, or mosque closest to the sampled geospatial coordinate. A chain referral process, wherein each household was asked about the next nearest Syrian household, was used to identify 20 to 30 households within each cluster.^10,11,23^ For multidwelling buildings, 1 household was randomly selected from the enumerated number of Syrian households. The next day, survey teams used the map to locate the first 15 households. To reduce selection bias, if households were absent, a follow-up appointment was made to revisit that same day. If households were unavailable after repeated attempts, they were replaced by the next mapped household. If 15 households could not be located, the cluster was considered complete.

### Data Collection and Variables

A questionnaire was designed in KoBoCollect software version 2.019.07 (KoboToolbox), which Jordanian nurses administered in Arabic using tablet computers. Each team consisted of 2 nurses. Nurses took a household census and enquired about prior diagnoses among adults aged 18 years or older to estimate prevalence in the adult population. To estimate biologically based prevalence among the higher-risk individuals aged 30 years or older and knowledge of relevant diagnoses, an adult aged 30 years or older was randomly selected. Nurses used auscultation and an electric sphygmomanometer to measure blood pressure (BP) 3 times, 5 minutes apart. Patients were seated with an unclothed arm supported at the level of his or her heart. A glucometer and testing strips were used to measure random blood glucose (RBG).Those with above-threshold BP and/or RBG were referred for care. An electronic weighing scale and measuring tape were used to measure body mass index (calculated as weight in kilograms divided by height in meters squared). Nurses asked about current medication and complications of disease, including heart problems, stroke, extremity numbness, poorly healing wounds, renal problems, and amputations (see eTable 1 in the Supplement for details).

An available adult aged 18 years or older with self-reported hypertension and/or diabetes was randomly selected to answer questions concerning access to care, current use and adherence to medication, and socioeconomic factors. Supervisors checked questionnaires before leaving the household. A 5-day training covered sampling, interviewing, role-playing, standardized biological measurement, and pilot testing of questionnaires in areas outside the sampling frame.

### Statistical Analysis

Analysis was conducted in Stata statistical software version 14.2 (StataCorp), using the *svyset* command to produce design effects and point estimates with appropriate 95% CIs. Two sets of prevalence estimates for hypertension, diabetes, and both conditions were calculated. Among adults aged 18 years or older, self-reported prevalence was calculated to estimate the known disease burden. This used the total number of self-reported diagnoses (numerator) and the size of the population aged 18 years or older (denominator).^11,24,25^ Among adults aged 30 years or older, above-threshold BP (mean of last 2 of 3 systolic BP and diastolic BP measures ≥140/90 mm Hg) and above-threshold RBG (≥200 mg/dL, regardless of fasting status; to convert blood glucose to mmol/L, multiply by 0.0555) were estimated.^24^ To estimate the total burden, above-threshold estimates were added to the number of respondents currently taking medication for each condition (numerator), along with the screened population size of adults aged 30 years or older (denominator) (World Health Organization STEPS method).^24^

To analyze age and sex as determinants of prevalence among adults aged 30 years or older, prevalence ratios (PRs) and 95% CIs were calculated using a generalized linear Poisson regression with robust variance.^26,27^ Multivariable logistic regression was used to investigate determinants (age and sex) of having an undiagnosed disease (ie, undiagnosed and positive result vs diagnosed regardless of screening result) achieving statistical significance (2-sided *P* < .05). For adults aged 18 years or older reporting hypertension and/or diabetes, sex-specific point estimates for access to care were calculated. Missing data are noted in the tables. Data analysis was performed from May to September 2019.

## Results

In total, 915 adults aged 30 years or older (mean [SD] age, 46.0 [12.8] years; 608 women [66.5%]) were available for the biological assessment, and 275 adults aged 18 years or older with self-reported hypertension and/or diabetes (mean [SD] age, 56.5 [13.2] years; 174 women [63.3%]) were available for the access to care interview. Fifteen of the 70 clusters had fewer than 15 households (range, 8-14 households). A total of 1025 households were visited and 1022 consented to participate. This represented 97% of the intended sample and 2798 adults aged 18 years or older. The mean (SD) household size was 6 (2.5) persons.

### Self-reported Diagnoses Among Adults Aged 18 Years or Older

Among adults aged 18 years or older, the self-reported prevalence of hypertension was 17.2% (95% CI, 15.9%-18.6%), that of diabetes (both insulin dependent and non–insulin dependent) was 9.8% (95% CI, 8.6%-11.1%), and that of both conditions was 7.3% (95% CI, 6.3%-8.5%). This equated to 1 in 5 adults reporting any diagnosis.

### Biological Assessment and Self-reported Diagnoses Among Adults Aged 30 Years or Older

Among adults aged 30 years or older screened, a total of 324 respondents (35.4%; 95% CI, 32.5%-38.4%) reported any diagnosis (Table 1); 286 respondents (31.3%; 95% CI, 28.6%-34.1%) reported having hypertension, 156 (17.1%; 95% CI, 14.6%-19.8%) reported having diabetes, and 118 (12.9%; 95% CI, 10.8%-15.3%) reported having both conditions. Similar proportions of men and women reported diagnoses of hypertension. More men than women reported ever smoking (196 men [63.8%; 95% CI, 58.7%-68.7%] vs 137 women [22.5%; 95% CI, 18.4%-27.3%]), and among the male smokers, most (182 men [92.9%]) were daily smokers. Among 324 persons with known diagnoses (35.4%), nearly all were taking medications (304 individuals [94.1%; 95% CI, 90.9%-96.2%]). Most of those with a diagnosis reported 1 or more complication (186 participants [57.4%; 95% CI, 51.5%-63.1%]).

**Table 1.** Health Profile of Adults Aged 30 Years or Older Assessed for Blood Pressure and Random Blood Glucose Level

Characteristic	Participants, No. (%) [95% CI]		
	Total (N = 915)	Men (n = 307)	Women (n = 608)
Among all 915 adults who were screened			
Age, mean (SD), y	46.0 (12.8)	46.3 (12.4)	45.9 (13.0)
Age group, y			
30-39	348 (38.0) [35.1-41]	110 (35.8) [31.1-40.8]	238 (39.1) [35.5-42.9]
40-59	404 (44.2) [41-47.3]	140 (45.6) [40.1-51.3]	264 (43.4) [39.8-47.2]
≥60	163 (17.8) [15.6-20.2]	57 (18.6) [15.2-22.5]	106 (17.4) [14.5-20.9]
Smoking			
Ever	333 (36.4) [32.8-40.2]	196 (63.8) [58.7-68.7]	137 (22.5) [18.4-27.3]
Daily^a^	278 (83.5) [79.0-87.2]	182 (92.9) [88.6-95.6]	96 (70.1) [60.8-77.9]
Reported being ever diagnosed			
Hypertension	286 (31.3) [28.6-34.1]	86 (28.0) [23.2-33.5]	200 (32.9) [29.6-36.4]
Diabetes	156 (17.1) [14.6-19.8]	41 (13.4) [10.3-17.2]	115 (18.9) [16-22.2]
Both conditions	118 (12.9) [10.8-15.3]	29 (9.5) [6.9-12.8]	89 (14.6) [12.1-17.6]
Total	324 (35.4) [32.5-38.4]	98 (31.9) [26.9-37.4]	226 (37.2) [33.8-40.7]
Among 324 adults with a known diagnosis (n = 98 men; n = 226 women)			
Take medication	304^b^ (94.1) [90.9-96.2]	93 (94.9) [88.1-97.9]	211^b^ (93.8) [90.2-96.1]
Complications			
Heart problems	91 (28.1) [23.8-32.8]	33 (33.7) [24.9-43.7]	58 (25.7) [20.7-31.4]
Stroke	25 (7.7) [5.1-11.6]	14 (14.3) [8.7-22.6]	11 (4.9) [2.5-9.2]
Numbness	127 (39.2) [34-44.7]	39 (39.8) [31.3-49]	88 (38.9) [33-45.2]
Unhealed sores	11 (3.4) [1.9-5.9]	3 (3.1) [1.0-9.1]	8 (3.5) [1.7-7.1]
Kidney failure	4 (1.2) [0.5-3.2]	2 (2.0) [0.5-7.9]	2 (0.9) [0.2-3.5]
Amputation	2 (0.6) [0.2-2.5]	1 (1.0) [0.1-7.2]	1 (0.4) [0.1-3.2]
Complication			
≥1	186 (57.4) [51.5-63.1]	65 (66.3) [56.6-74.8]	121 (53.5) [46.8-60.2]
≥2	63 (19.4) [15.4-24.3]	21 (21.4) [15.2-29.4]	42 (18.6) [13.8-24.6]

^a^ Percentages of daily smokers are calculated using the number of ever smokers as the denominator.

^b^ One woman refused to discuss medications (total, 323 respondents; 225 women).

Table 2 shows the screening results. The proportion of participants with above-threshold BP was 24.0% (95% CI, 21.0%-27.3%; 220 respondents); 86 of 220 participants with above-threshold BP did not report knowing their diagnosis (39.1%; 95% CI, 32.9%-45.6%). The proportion of participants with above-threshold RBG was 10.7% (95% CI, 8.7%-13.1%; 96 participants); 22 of 96 participants with above-threshold RBG did not report knowing their diagnosis (22.9%; 95% CI, 15.7%-32.1%). Body mass index was classified as overweight for 273 participants (30.1%; 95% CI, 27.1%-33.3%) and as obese for 478 participants (52.7%; 95% CI, 49.2%-56.2%), for a total of 751 participants who were overweight or obese (82.8%; 95% CI, 79.7%-85.5%).

**Table 2.** Screening Results for Adults Aged 30 Years or Older Assessed for Blood Pressure and Random Blood Glucose Level

Characteristic	Participants, No. (%) [95% CI]		
	Total (N = 915)	Men (n = 307)	Women (n = 608)
Blood pressure			
Systolic, mean (SD), mm Hg	123.7 (15.8)	124.5 (15)	123.3 (16.2)
Diastolic, mean (SD), mm Hg	79.2 (10.1)	80 (9.1)	78.8 (10.6)
≥140/90 mm Hg	220 (24.0) [21.0-27.3]	67 (21.8) [16.7-28]	153 (25.2) [21.8-28.9]
Without known diagnosis^a^	86 (39.1) [32.9-45.6]	33 (49.3) [37.2-61.4]	53 (34.6) [27.6-42.5]
Random blood glucose, mean (SD), mg/dL^b^	142.2 (59.5)	140.9 (64.6)	142.8 (56.6)
≥200 mg/dL	96 (10.7) [8.7-13.1]	33 (10.8) [7.6-15.2]	63 (10.6) [8.3-13.6]
Without known diagnosis^c^	22 (22.9) [15.7-32.1]	9 (27.3) [15.4-43.7]	13 (20.6) [12.7-31.7]
Increased blood pressure and random blood glucose	36 (4.0) [2.7-5.9]	12 (3.9) [2.1-7.3]	24 (4.1) [2.6-6.4]
Body mass index^d^^,^^e^			
Mean (SD)	31 (6.6)	28.7 (5.6)	32.1 (6.7)
Categories			
Underweight, <18.5	9 (1.0) [0.5-1.8]	7 (2.3) [1.1-4.6]	2 (0.3) [0.1-1.4]
Normal, 18.5-24.9	147 (16.2) [13.7-19.1]	74 (24.1) [19.1-30]	73 (12.2) [9.9-14.9]
Overweight, 25.0-29.9	273 (30.1) [27.1-33.3]	110 (35.8) [29.9-42.2]	163 (27.2) [24-30.6]
Obese, ≥30.0	478 (52.7) [49.2-56.2]	116 (37.8) [32.2-43.7]	362 (60.3) [56.5-64]
Total obese or overweight	751 (82.8) [79.7-85.5]	226 (73.6) [67.2-79.2]	525 (87.5) [84.8-89.8]

SI conversion factor: To convert blood glucose to mmol/L, multiply by 0.0555.

^a^ Percentages of patients without a known diagnosis are calculated using the number of patients with blood pressure greater than or equal to 140/90 mm Hg as the denominator.

^b^ Eighteen persons refused random blood glucose screening (denominator = 897).

^c^ Percentages of patients without a known diagnosis are calculated using the number of patients with random blood glucose level greater than or equal to 200 mg/dL as the denominator.

^d^ Body mass index is calculated as weight in kilograms divided by height in meters squared.

^e^ Eight persons refused body measurements for body mass index (denominator = 907).

### Prevalence and Its Determinants Among Adults Aged 30 Years or Older

The prevalence of hypertension was 39.5% (95% CI, 36.4%-42.6%; 361 participants), that of diabetes was 19.3% (95% CI, 16.7%-22.1%; 173 participants), and that of both conditions was 13.5% (95% CI, 11.4%-15.9%; 121 participants) (Table 3). When adjusted for age and sex, prevalence across conditions increased with age. For hypertension, prevalence increased from 13.2% (age 30-39 years) to 45.5% (age 40-59 years; adjusted PR [aPR], 3.5%; 95% CI, 2.6%-4.6%) to 80.4% (age ≥60 years, aPR, 6.1%; 95% CI, 4.6%-8.1%). A similar magnitude of increase was seen for diabetes and both conditions. Compared with men, women had a higher prevalence of diabetes (aPR, 1.3%; 95% CI, 1.0%-1.7%) and of both conditions (aPR, 1.4%; 95% CI, 1.0%-2.0%), but neither difference was significant. When adjusted for age and sex, the prevalence of obesity or overweight increased with age. Women (aPR, 1.2%; 95% CI, 1.1%-1.3%) and those with any diagnosis had a higher prevalence of obesity or overweight.

**Table 3.** Prevalence and PRs for Hypertension, Diabetes, Both Conditions, and Obese or Overweight Status

Characteristic	Participants, No.	Prevalence, % (95% CI)	PR (95% CI)		*P* value for adjusted PR
			Unadjusted	Adjusted	
Hypertension	361	39.5 (36.4-42.6)	NA	NA	NA
Age group, y					
30-39	46	13.2 (10.2-17)	1 [Reference]	1 [Reference]	NA
40-59	184	45.5 (41.4-49.8)	3.4 (2.6-4.6)	3.5 (2.6-4.6)	<.001
≥60	131	80.4 (73.2-86.0)	6.1 (4.6-8)	6.1 (4.6-8.1)	<.001
Sex					
Male	116	37.8 (32.2-43.8)	1 [Reference]	1 [Reference]	NA
Female	245	40.3 (36.6-44.2)	1.1 (0.9-1.3)	1.1 (0.9-1.3)	.20
Diabetes^a^	173	19.3 (16.7-22.1)	NA	NA	NA
Age group, y					
30-39	17	5 (3.1-8)	1 [Reference]	1 [Reference]	NA
40-59	88	22.1 (18.3-26.3)	4.4 (2.7-7.2)	4.4 (2.7-7.3)	<.001
≥60	68	42.8 (35.2-50.6)	8.5 (5.2-14)	8.6 (5.2-14.1)	<.001
Sex					
Male	50	16.4 (12.7-20.9)	1 [Reference]	1 [Reference]	NA
Female	123	20.8 (17.7-24.3)	1.3 (0.9-1.7)	1.3 (1-1.7)	.07
Both conditions^a^	121	13.5 (11.4-15.9)	NA	NA	NA
Age group, y					
30-39	6	1.8 (0.7-4.3)	1 [Reference]	1 [Reference]	NA
40-59	58	14.5 (11.6-18.1)	8.2 (3.6-18.8)	8.3 (3.6-19)	<.001
≥60	57	35.9 (28.5-44)	20.3 (8.9-46.3)	20.4 (9-46.3)	<.001
Sex					
Male	33	10.8 (7.9-14.7)	1 [Reference]	1 [Reference]	NA
Female	88	14.9 (12.3-17.9)	1.4 (0.9-2)	1.4 (1-2)	.05
Obese and overweight^b^	751	82.8 (79.7-85.5)	NA	NA	NA
Age group, y					
30-39	262	75.3 (70-80)	1 [Reference]	1 [Reference]	NA
40-59	347	86.5 (82.3-89.9)	1.1 (1.1-1.2)	1.1 (1-1.2)	.001
≥60	142	89.9 (83.4-94)	1.2 (1.1-1.3)	1.1 (1-1.2)	.002
Sex					
Male	226	73.6 (67.2-79.1)	1 [Reference]	1 [Reference]	NA
Female	525	87.5 (84.8-89.8)	1.2 (1.1-1.3)	1.2 (1.1-1.3)	<.001
Diagnosis					
No diagnosis	455	77.5 (73.5-81)	1 [Reference]	1 [Reference]	NA
Hypertension	146	89 (82.2-93.4)	1.1 (1.1-1.2)	1.1 (1-1.2)	.01
Diabetes	37	97.4 (82.8-99.7)	1.3 (1.2-1.3)	1.2 (1.1-1.3)	<.001
Both conditions	113	95.8 (90.2-98.2)	1.2 (1.2-1.3)	1.2 (1.1-1.2)	<.001

Abbreviations: NA, not applicable; PR, prevalence ratio.

^a^ Eighteen persons refused to undergo random blood glucose screening (denominator = 897).

^b^ Eight persons refused body measurements for body mass index (denominator = 907).

When 86 undiagnosed persons with above-threshold BP (ie, undetected without treatment) were compared with 286 patients with diagnosed hypertension, older age groups showed decreased odds of not having a diagnosis (age 40-59 years, adjusted odds ratio, 0.3; 95% CI, 0.1-0.5; *P* < .001; age ≥60 years, adjusted odds ratio, 0.1; 95% CI, 0.1-0.3; *P* < .001). No associations were shown for above-threshold RBG.

### Access to Care Among Adults Aged 18 Years or Older With Hypertension and/or Diabetes

A total of 275 adults aged 18 years or older who reported a diagnosis of hypertension and/or diabetes were asked about access to care (eTable 2 in the Supplement shows patient demographic characteristics). Nearly all were registered with United Nations High Commissioner for Refugees (96.7%; 95% CI, 94.2%-98.2%) and the government (91.6%; 95% CI, 87.7%-94.4%). The mean (SD) time living in the current residence was 2.8 (2.1) years. Most lived in apartments or houses (92.7%; 95% CI, 87.2%-96.0%) and had electricity (>99%) and access to a refrigerator for insulin (94.9%; 95% CI, 91.5%-97.0%). Respondents were closely distributed in terms of education: none, 33.1% (95% CI, 27.1%-39.7%), primary education, 29.5% (95% CI, 24.3%-35.2%), and secondary education, 34.6% (95% CI, 28.2%-41.5%). The primary source of household funding during the last month was humanitarian assistance (70.6%; 95% CI, 65%-75.6%), including vouchers from the World Food Programme (48.4%; 95% CI, 41.9%-54.9%) and cash assistance from NGOs (22.2%; 95% CI, 17.6%-27.6%).

Table 4 outlines access to care among adults aged 18 years or older who reported a diagnosis of hypertension, diabetes, or both conditions; 100 individuals (36.4%; 95% CI, 31.1%-41.9%) had 2 or more complications. Commonly reported complications were numbness (peripheral neuropathy, 123 respondents [44.7%; 95% CI, 39.7%-49.9%]), heart problems (86 respondents [31.3%; 95% CI, 25.1%-38.2%]), and eye problems (72 respondents [26.2%; 95% CI, 21.0%-32.1%]). Nearly all reported taking medication (265 respondents [96.4%; 95% CI, 93.1%-98.1%]). During the past week, 71 respondents (26.8%; 95% CI, 21.3%-33.1%) reported missing a dose because of forgetting (23 of 71 respondents [32.4%; 95% CI, 22.8%-43.7%]), feeling they did not need it (19 of 71 respondents [26.8%; 95% CI, 17.8%-38.1%]), or cost (16 of 71 respondents [22.5%; 95% CI, 13.6%-35.0%]). The median (interquartile range) total days of medication missed during the month before the survey was 8.7 (2-10) days, and 47 of 265 respondents currently taking medication (17.7%; 95% CI, 12.7%-24.2%) reported taking a smaller dose to prolong their supply, in the past month.

**Table 4.** Access to Care for Adults Aged 18 Years or Older With Diagnoses of Hypertension, Diabetes, or Both Conditions

Variable	Participants, No. (N = 275)	Prevalence, % (95% CI)
Diagnosed complications		
Heart problems	86	31.3 (25.1-38.2)
Numbness	123	44.7 (39.7-49.9)
Eye problem	72	26.2 (21-32.1)
Stroke	29	10.6 (7.4-14.8)
Diabetic foot	19	6.9 (4.4-10.7)
Kidney failure	5	1.8 (0.8-4.3)
Complications		
1	79	28.7 (24.2-33.8)
≥2	100	36.4 (31.1-41.9)
Factors associated with poor access		
Poor self-rated health status	96	34.9 (28.4-42.1)
Disability preventing travel to clinic	75	27.3 (22.8-32.2)
Access to care		
Sought outpatient care (last 30 d)	135	49.1 (43.3-54.9)
Received outpatient care	116	85.9 (77.7-91.5)
If did not receive care sought, why not (n = 19)^a^		
Cost	14	73.7 (49.7-88.8)
Forgot	1	5.3 (0.7-30.7)
Problem with facility services	1	5.3 (0.5-36.7)
No transportation	1	5.3 (0.5-36.7)
Last place sought care		
Clinic		
Nongovernmental organization	118	42.9 (36.3-49.8)
International Rescue Committee	95	34.6 (28.6-41.0)
Private clinic	27	9.8 (6.5-14.7)
Government clinic	25	9.1 (5.9-13.8)
Medication		
Currently taking medication	265	96.4 (93.1-98.1)
Took less dose (last 30 d)	47	17.7 (12.7-24.2)
Days of medication missed (last 30 d), median	8.7	6.1-11.3
Missed a dose (last 7 d)	71	26.8 (21.3-33.1)
If missed a dose, why (n = 71)^a^		
Forgot	23	32.4 (22.8-43.7)
Felt did not need medication	19	26.8 (17.8-38.1)
Cost	16	22.5 (13.6-35.0)
Wait times or supply at facility	4	5.6 (2.2-13.7)

^a^ Only major responses are listed, and percentages may not add up to 100%.

Respondents reported last receiving care at the International Rescue Committee clinic (95 respondents [34.6%; 95% CI, 28.6%-41.0%]), other NGO clinic (118 respondents [42.9%; 95% CI, 36.3%-49.8%]), private clinic (27 respondents [9.8%; 95% CI, 6.5%-14.7%]), or government clinic (25 respondents [9.1%; 95% CI, 5.9%-13.8%]). Nearly one-half reported the need to seek outpatient care in the last 30 days (135 respondents [49.1%; 95% CI, 43.3%-54.9%]). The majority of this group (116 respondents [85.9%; 95% CI, 77.7%-91.5%]) successfully obtained care. Of the 19 respondents (14.1%) who tried but did not receive care, the main barrier was costs (14 respondents [73.7%; 95% CI, 49.7%-88.8%]); because primary care is free, this cost includes travel, supplies, and lost time. Costs for services and out-of-pocket costs were the main barriers for referral (54.6%), specialist care (84%), and laboratory testing (95.2%).

## Discussion

This cross-sectional study documents the burden of hypertension and diabetes and access to care among Syrian refugees displaced for many years in northern Jordan. The biologically based rates of hypertension (39.5%), diabetes (19.3%), and both conditions (13.5%) among adults aged 30 years or older were moderately higher than rates of self-reported diagnoses (31.3% for hypertension, 17.1% for diabetes, and 12.9% for both), suggesting an awareness of morbidity and access to diagnosis. Although comparative data are scarce, Turkey’s 2017 national survey of Syrian refugees demonstrated lower hypertension prevalence among adults aged 30 years or older (31.5%).^28^ The high prevalence of having at least 1 complication (57.4%) among patients highlights the risk of worsening disease. Similarly, the exceptionally high prevalence of obesity and overweight among adults aged 30 years or older (82.8%), particularly among women, highlights opportunities for basic primary prevention to avoid disease development and progression. Despite nearly all patients reporting taking medication, almost one-half of patients aged 18 years or older did not seek care during the month before the survey and nearly one-third missed their medications. This highlights that continuous management and adherence to medication is fragile. More than two-thirds of households were dependent on humanitarian assistance to cover their basic needs, of which adult health care is one of many. Rehr et al^11^ reported that 79.3% of Syrian households in northern Jordan were in debt, and previous studies^29,30^ have identified costs for travel, laboratory testing, referral, as critical barriers to continuous care.

### Implications

With 94.1% of adults aged 30 years or older with a diagnosis taking medication, these data suggest that diagnosis and basic management are achievable for these urban refugees. The reported use of medication, however, represents self-reported access at 1 point in time rather than long-term access or adherence. Without complementary strategies, basic management may diminish the concept of care to mainly drug management.^5,31^ Certain risk groups should be targeted by primary and secondary prevention to avoid severe disease, complications, and mortality. First, these data suggest that women have higher odds of being obese or overweight, diabetic, and having both conditions, as do persons aged 40 years or older. Second, persons aged 30 to 39 years had higher odds of having undiagnosed conditions and having above-threshold BP and, thus, present as a different risk group requiring targeted intervention. Diagnosing hypertension when patients are young may allow for concomitant diabetes to be identified and controlled at an early stage. Among patients with known diagnoses, there is a critical need to treat complications to prevent severe disease and avoid excessive health care costs. This is important given the prolonged nature of displacement globally and limited resources for refugee health.^32^

Population-based primary prevention policies targeting diet, smoking, and physical activity in Jordan should consider the socioeconomic environments of refugees.^33^ However, NGOs and district health systems could also sharpen their focus on high-risk groups. First, this includes broadening the group considered to be at risk of developing NCDs to age 30 years and older, especially women, during clinic-based screening. Second, given the poor access to secondary care, primary care must improve awareness among practitioners of early detection and management of simple complications (eg, diabetic foot), similar to primary care approaches in Iran.^17,29,30^ Third, high-risk patients (eg, with poorly controlled disease, serious complications, or type 1 diabetes) could be counseled on both medical and preventative interventions to reduce their risk of deterioration.^7^ This includes self-management protocols for monitoring and recognition of danger signs, exercise classes for women in private spaces, and counseling on reducing salt intake.^12,34,35^ Fourth, psychosocial care related to conflict and displacement can reduce hopelessness and increase motivation for seeking care.^29,36^ Given the presence of CHW networks in protracted crises, task-shifting of nonclinical activities, such as routine monitoring, patient education, and psychosocial and peer support to CHWs, would relieve staff.^7,16,34,37^

### Limitations

This study has limitations that should be addressed. The chain referral method introduces selection bias because it is dependent on the respondents’ knowledge of neighboring Syrian households and possible predilection to refer to family or friends. Unhoused persons and refugees who are intentionally hidden are excluded. Nonetheless, nearly all clusters had sufficient households, and data collectors were indeed referred to persons living in tents, shacks, and empty buildings. Two-thirds of respondents were female, and the health of working men may be different from that of those available during the daytime. The biological assessments cannot be used to confirm diagnoses. Estimation of diabetes prevalence is a known problem for surveys.^38^ The use of RBG instead of fasting blood glucose is nonstandard; therefore, prevalence is approximated.^24^

## Conclusions

This study documents the impact of long-term displacement on Syrians who sought refuge in Jordan for a duration long enough where achieving disease control should become feasible.^7^ By focusing programs on early identification through clinical screening and improving adherence to continuous care and secondary prevention among patients, severe morbidity among refugees could be minimized here and in other protracted crises.

## Notes

Supplement. **eTable 1.** Biological Measurement Methods, Classification of Medications, and Classification of Complications **eTable 2.** Demographics for 275 Adults 18 Years or Older With Diagnoses of Hypertension, Diabetes, or Both Conditions
