# Supplementary material for: Access to Care and Prevalence of Hypertension and Diabetes Among Syrian Refugees in Northern Jordan
Source: JAMA Netw Open. 2020 Oct 14;3(10):e2021678. doi: 10.1001/jamanetworkopen.2020.21678 (PMC7557515; doi:10.1001/jamanetworkopen.2020.21678)
Supplement: Supplement. — eTable 1. Biological Measurement Methods, Classification of Medications, and Classification of Complications eTable 2. Demographics for 275 Adults 18 Years or Older With Diagnoses of Hypertension, Diabetes, or Both Conditions [file jamanetwopen-e2021678-s001.pdf]

Supplemental Online Content

Ratnayake R, Rawashdeh F, AbuAlRub R, et al. Access to care and prevalence of hypertension and diabetes among Syrian refugees in northern Jordan. *JAMA Netw Open*. 2020;3(10):e2021678. doi:10.1001/jamanetworkopen.2020.21678

eTable 1. Biological Measurement Methods, Classification of Medications, and Classification of Complications

eTable 2. Demographics for 275 Adults 18 Years or Older With Diagnoses of Hypertension, Diabetes, or Both Conditions

This supplemental material has been provided by the authors to give readers additional information about their work.

© 2020 Ratnayake R et al. *JAMA Network Open.*

eTable 1. Biological Measurement Methods, Classification of Medications, and Classification of Complications

Biological measure	Method and/or responses
Blood pressure (BP)	- BP was measured using auscultation and electric sphygmomanometers<br>- Patients were seated with unclothed arms supported at the level of their heart<br>- BP was measured 3 times 5 minutes apart<br>- Respondents with above-threshold BP (>140/90 mmHg) were referred for care<br>- Respondents with lowest BP >180/110 mmHg were checked for symptoms of heart attack or stroke. If no symptoms, they were refer urgently to nearby clinic. If symptoms, referred immediately.
Random blood glucose (RBG)	- RBG was measured using a fingerstick to obtain a pinprick of blood on a test strip, which was measured with a glucometer<br>- Respondents were asked: when was the last time you ate or drank anything except water?<br>- Those with above-threshold RBG were referred for care (if fasting blood glucose >126 mg/dL or RBG ≥200 mg/dL)
Medication use	If a respondent reported a diagnosis of hypertension and/or diabetes they were asked the following:<br><br>Are you currently taking medications (including insulin) for any of the following? [select all that apply]<br>1. Diabetes<br>2. High blood pressure<br>3. Both diabetes and high blood pressure<br>4. I do not take medications for either diabetes or high blood pressure
Complications	Have you ever had any of the following? [select all that apply]<br>1. Heart attack and/or heart problems<br>2. Stroke<br>3. Numbness in your feet<br>4. Sores on your feet that do not heal<br>5. Kidney failure and/or dialysis<br>6. Amputation

© 2020 Ratnayake R et al. *JAMA Network Open.*

eTable 2. Demographics for 275 Adults 18 Years or Older With Diagnoses of Hypertension, Diabetes, or Both Conditions

	N	% (95% CI)
**Sex**		
Male	101	36.7 (30.6-43.4)
Female	174	63.3 (56.7-69.4)
**Age group (years)**		
18-29	2	0.7 (0.2-2.9)
30-59	158	57.5 (51.3-63.4)
≥60	115	41.8 (35.9-48)
**Registration**		
Ministry of Interior	252	91.6 (87.7-94.4)
United Nations High Commissioner for Refugees	266	96.7 (94.2-98.2)
**Type of housing**		
Apartment or house	255	92.7 (87.2-96)
Temporary residence (unfinished, tent, shack, public building)	20	7.3 (4-12.8)
**Electricity available**	274	99.6 (97.4-100)
**Refrigeration available**	261	94.9 (91.5-97)
**Educational level**		
No school	91	33.1 (27.1-39.7)
Primary	81	29.5 (24.3-35.2)
Secondary school	95	34.6 (28.2-41.5)
Post-secondary	8	2.9 (1.4-6)
**Primary funds (last month)**[a]		
Humanitarian assistance	194	70.6 (65-75.6)
World Food Program food vouchers	133	48.4 (41.9-54.9)
Cash transfers	61	22.2 (17.6-27.6)
Sale of assistance	15	5.5 (3.2-9.2)
Daily labor/paid work	59	21.5 (16.7-27.1)
Loans, remittances, gifts	6	2.2 (1-4.7)

[a]1 response refused (N=274)

© 2020 Ratnayake R et al. *JAMA Network Open.*
